# Nutritional paradoxes of the new Dietary Guidelines for Americans (2025–2030): a critical analysis

**DOI:** 10.1590/2175-8239-JBN-2026-PO01en

**Published:** 2026-03-23

**Authors:** Carla Maria Avesani, Lilian Cuppari

**Affiliations:** 1 Karolinska Institutet, Department of Clinical Science, Intervention and Technology, Division of Renal Medicine, Baxter Novum, Stockholm, Sweden.; 2 Universidade Federal de São Paulo, Departamento de Medicina, Disciplina de Nefrologia, São Paulo, SP, Brazil.

**Keywords:** Food, Diet, Nutritional Sciences, Food Guide

## Abstract

In January 2026, the new Dietary Guidelines for Americans were released, generating substantial international debate. Unlike previous editions, these guidelines were not primarily based on the report of the Dietary Guidelines Advisory Committee but on an independent scientific review, a process that raised concerns regarding transparency and potential conflicts of interest. This article critically examines the main recommendations of the new Dietary Guidelines for Americans, with particular emphasis on their implications for metabolic, cardiovascular, and kidney health. While the guidelines appropriately emphasize the reduction of ultra-processed food consumption and the promotion of minimally processed foods, several inconsistencies with contemporary scientific evidence are identified. Key concerns include the use of an outdated inverted food pyramid, a strong emphasis on high-protein intake (1.2–1.6 g/kg/day) predominantly from animal sources, and an internal contradiction between recommended protein intake and limits on saturated fat consumption. The proposed dietary pattern may also inadequately support gut microbiota health due to relatively low recommendations for fruits, vegetables, and whole grains. These issues may also be relevant to nephrology, given the growing popularity of high-protein diets and their potential renal implications. Overall, while the guidelines contain positive elements, their scientific coherence and alignment with established evidence-based dietary patterns remain questionable, potentially limiting their effectiveness as a public health tool.

## Introduction

On January 7, 2026, the new Dietary Guidelines for Americans (DGA) were published, triggering extensive discussion in the international health and nutrition media^1^. The guidelines consist of: (i) a concise printed document intended for the general population^1^; (ii) a short institutional video (approximately 1 minute and 25 seconds – https://realfood.gov); (iii) a scientific report with appendices^2,3^; and (iv) an appendix detailing daily food group servings according to different levels of energy intake^4^.

A central aspect of this process was the decision not to use, as the primary scientific basis, the report produced by the Dietary Guidelines Advisory Committee (DGAC), published in December 2024 and originally intended to inform the development of the guidelines^5^. The rejection of the DGAC report was justified by alleged methodological and conceptual limitations, including the use of a health-equity-centered framework. Instead, the responsible administration chose to conduct an independent review of the scientific evidence, relying on the expertise of a newly appointed scientific committee and arguing that the guidelines should prioritize the best available evidence in nutrition science, with a direct focus on the prevention and reversal of chronic diseases and the promotion of metabolic health^2^. It is noteworthy that some experts involved in this new review reported previous or current financial ties to the meat, dairy, supplement, and infant formula industries^6^. Although such relationships do not necessarily imply scientific bias, their presence raises concerns regarding potential conflicts of interest in the formulation of the recommendations presented in the guidelines.

## What does the New Guide Propose?

The new guidelines adopt the slogans “Make America Healthy Again” and “Eat Real Food,” grounding their message in the epidemiological reality of the United States, where approximately 70% of the population suffers from overweight or obesity, with substantial implications for the burden of chronic non-communicable diseases^1^. The document attributes this scenario largely to the high consumption of ultra-processed foods—rich in refined carbohydrates, added sugars, fats, sodium, and chemical additives—combined with low levels of physical activity.

At this point, the guidelines convey a relevant and well-supported message: the need to reduce ultra-processed food consumption and prioritize minimally processed foods^7^. However, as the document progresses, several recommendations emerge whose consistency with contemporary scientific evidence appears less robust.

## Food Pyramid Graphic: an Outdated and Confusing Model

One of the most controversial elements of the new guidelines is the use of an inverted food pyramid as the primary graphical representation. At the base of the pyramid, occupying half of the space, are animal-source foods—including high-fat red meat, poultry, eggs, fish, and whole-fat dairy products—as well as butter. The other half of the base consists of fruits, vegetables, and legumes. Whole grains are placed at the narrow top of the pyramid.

The use of food pyramids as a population-level guidance tool is widely regarded as outdated, as it oversimplifies the complexity of human diets and fails to reflect current scientific understanding of dietary patterns, health, and chronic disease^8^. Pyramid models emphasize quantities and portions of food groups, whereas contemporary evidence highlights the importance of food quality and overall dietary patterns as more relevant determinants of health^8^. Moreover, pyramids do not adequately capture food combinations, cultural diversity, or regional dietary practices—key features of modern dietary guidelines such as the Brazilian Dietary Guidelines^9^ and the Nordic Nutrition Recommendations^10^.

Accordingly, many countries, including Brazil, have replaced pyramids with plate-based models that more effectively illustrate balanced and culturally adapted dietary patterns. In the case of the U.S. guidelines, the adoption of an inverted pyramid further exacerbates the limitations of this model, making its interpretation less intuitive and potentially confusing for the general population.

## Other Key Recommendations

The document also outlines the following general recommendations^1^: (1) consume an appropriate amount of food for individual needs; (2) prioritize protein-rich foods at every meal; (3) consume dairy products; (4) eat fruits and vegetables throughout the day; (5) incorporate healthful fats; (6) focus on whole foods; (7) limit ultra-processed foods, added sugars, and refined carbohydrates; (8) limit alcoholic beverage consumption; and (9) provide specific guidance for population subgroups, including infants and young children, school-aged children, adolescents, young adults, pregnant and lactating women, older adults, individuals with chronic diseases, vegetarians, and vegans.

## Emphasis on Protein intake: What is the Scientific Evidence?

Among these recommendations, the emphasis on protein intake is particularly striking. The guidelines suggest intakes ranging from 1.2 to 1.6 g/kg/day, to be achieved predominantly through animal protein sources such as red meat, poultry, eggs, and whole-fat dairy products—foods that are well known to contain high amounts of saturated fat.

This recommendation substantially exceeds the protein intake recommended by the U.S. Recommended Dietary Allowance (RDA)^11^ and by the World Health Organization^12^, both of which set protein needs for healthy adults at approximately 0.8 g/kg/day. While individuals with very high levels of physical activity may require greater protein intake, for the majority of adults—who are largely sedentary—excess protein consumption may contribute to a positive energy balance, thereby promoting weight gain and obesity.

Furthermore, prioritizing animal protein contrasts with a growing and consistent body of evidence from the past 10–15 years showing that predominantly plant-based dietary patterns—where 50–80% of protein intake is derived from plant sources—provide a more favorable nutrient and fiber profile and are associated with a reduced risk of chronic non-communicable diseases, including cardiovascular disease and chronic kidney disease^13^. This issue is particularly relevant to the nephrology community, given the increasing popularity of high-protein diets among young adults seeking muscle mass gain, which may induce glomerular hyperfiltration, although long-term adverse renal outcomes remain inconclusive^14^.

The environmental implications of this recommendation are also substantial. In October 2025, an updated EAT–Lancet dietary framework was published, proposing a dietary pattern that simultaneously promotes human health and environ­mental sustainability by prioritizing plant-based foods and limiting animal-source and ultra-processed foods^15^. This evidence-based framework highlights the environmental and health risks associated with diets high in animal protein.

Taken together, these considerations indicate that the emphasis on protein intake in the new U.S. Dietary Guidelines stands in clear opposition to many contemporary dietary guidelines worldwide.

## Internal Inconsistency: Saturated Fat Versus the Proposed Dietary Pattern

Another critical issue is the internal inconsistency of the guidelines. While they recommend prioritizing protein-rich foods at every meal—illustrated by images of high-fat meats in the inverted pyramid and by a daily protein intake of 1.2–1.6 g/kg—the guidelines simultaneously advise limiting saturated fat intake to less than 10% of total daily energy. In addition, although oils rich in mono- and polyunsaturated fatty acids, such as olive oil, are recommended, butter and animal fat are presented as possible alternatives.

This combination of recommendations creates a dietary paradox that undermines the practical applicability of the guidelines. From a dietary planning perspective, it is difficult—if not impossible—to reconcile a diet rich in meat and whole-fat dairy products, with the optional use of butter or animal fat as alternatives to olive oil, while still maintaining saturated fat intake below the recommended threshold.

## Fruits, Vegetables, Whole Grains, and the Gut Microbiota

The quantitative recommendations for fruits and vegetables—two servings of fruit and three servings of vegetables per day—are lower than those suggested by dietary patterns consistently associated with a reduced risk of obesity and chronic diseases. Similarly, positioning whole grains at the narrow top of the pyramid, with a recommendation of only two to four servings per day, diverges from widely endorsed healthy dietary models.

Collectively, the proposed amounts of meat, dairy, fruits, vegetables, whole grains, and fats are unlikely to support a healthy gut microbiota, despite the guidelines’ acknowledgment of its importance for health. An emphasis on animal-source foods combined with relatively low intake of dietary fiber from fruits, vegetables, legumes, and whole grains may reduce gut microbial diversity and the production of beneficial metabolites such as short-chain fatty acids^16^. This dietary pattern may promote low-grade chronic inflammation and disrupt the gut–heart and gut–kidney axes, mechanisms implicated in the development of metabolic, cardiovascular, and renal diseases^16^.

## Positive Aspects

Despite these criticisms, the guidelines present some positive elements. The central message of “eating real food” has the potential to reduce ultra-processed food consumption and encourage home cooking, which may yield health benefits over time. Recommendations to avoid refined grains with added sodium, sugar, fats, and chemical additives, as well as to limit sugar-sweetened beverages and alcohol intake, are also well supported by robust evidence. Additionally, the simplified material developed for the general population, written in accessible language, represents a positive feature of the guidelines.

## Relevance for the Brazilian Population

In the Brazilian context, the impact of these guidelines is negligible. The Brazilian Dietary Guidelines, published in 2014^9^, are well adapted to the country’s dietary, socioeconomic, and cultural realities and are internationally recognized as one of the most innovative food-based dietary guidelines worldwide. They emphasize regional culinary traditions, minimally processed foods, seasonality, and cultural practices and were pioneering in explicitly incorporating healthy and just food systems, including environmental sustainability and equity in food production and access.

## Conclusion

In summary, the new Dietary Guidelines for Americans introduce important advances by recognizing the central role of minimally processed foods and emphasizing the reduction of ultra-processed food consumption in a population burdened by obesity and chronic diseases. However, the guidelines also reveal significant internal inconsistencies, particularly regarding the excessive emphasis on animal protein intake, the use of an outdated and inverted food pyramid as the main graphical tool, and the conflict between recommended protein intake levels and limits on saturated fat consumption.

By moving away from contemporary models based on integrated, sustainable dietary patterns aligned with metabolic, cardiovascular, kidney, and environmental health, the guidelines risk limiting their effectiveness as a public health instrument. Given the strategic role of dietary guidelines in shaping food systems, policies, and individual choices, scientific coherence, practical feasibility, and alignment with consolidated evidence are essential. In this regard, the new U.S. guidelines raise more questions than answers and underscore the importance of transparent and scientifically rigorous processes in the development of population-level dietary recommendations.

## Data Availability

No new data were generated or analyzed in this study.
