# Supplementary material for: Nutritional paradoxes of the new Dietary Guidelines for Americans (2025–2030): a critical analysis
Source: J Bras Nefrol. 2026 Mar 23;48(2):2026PO01. doi: 10.1590/2175-8239-JBN-2026-PO01en (PMC13008419; doi:10.1590/2175-8239-JBN-2026-PO01en)
Supplement: Translation

# Translation: Paradoxos nutricionais das novas Diretrizes Alimentares dos Estados Unidos (2025–2030): uma análise crítica

**DOI:** 10.1590/2175-8239-JBN-2026-PO01pt

**Authors:** Carla Maria Avesani, Lilian Cuppari

**Affiliations:** 1 Karolinska Institutet, Department of Clinical Science, Intervention and Technology, Division of Renal Medicine, Baxter Novum, Stockholm, Sweden.; 2 Universidade Federal de São Paulo, Departamento de Medicina, Disciplina de Nefrologia, São Paulo, SP, Brasil.

**Keywords:** Alimentos, Dieta, Ciências da Nutrição, Guias Alimentares

## Abstract

Em janeiro de 2026, foram publicadas as novas Diretrizes Alimentares para Americanos, gerando amplo debate internacional. Diferentemente de edições anteriores, essas diretrizes não se basearam prioritariamente no relatório do *Dietary Guidelines Advisory Committee*, mas em uma revisão científica independente, pro­cesso que levantou preocupações quanto à transparência e a possíveis conflitos de interesse. Este artigo analisa criticamente as principais recomendações das novas Diretrizes Alimentares para os Americanos, com ênfase em suas implicações para a saúde metabólica, cardiovascular e renal. Embora as diretrizes enfatizem adequadamente a redução do consumo de alimentos ultraprocessados e a promoção de alimentos minimamente processados, são identificadas diversas inconsistências em relação às evidências científicas contemporâneas. Destacam-se o uso de uma pirâmide alimentar invertida e desatualizada, a forte ênfase na ingestão elevada de proteínas (1,2–1,6 g/kg/dia), predominantemente de origem animal, e a contradição interna entre a recomendação de ingestão proteica elevada e a limitação do consumo de gorduras saturadas. O padrão alimentar proposto também pode ser inadequado para a saúde da microbiota intestinal, devido às recomendações rela­tivamente baixas de frutas, verduras e grãos integrais. Esses aspectos podem também ser relevantes para a nefrologia, considerando a crescente popularidade de dietas hiperproteicas e seus potenciais efeitos renais. Em conjunto, embora as diretrizes apresentem pontos positivos, sua coerência científica e seu alinhamento com padrões alimentares baseados em evidências consolidadas perma­necem questionáveis, o que pode limitar sua efetividade como instrumento de saúde pública.

## Introdução

No dia 7 de janeiro de 2026, foram publicadas as novas Diretrizes Alimentares dos Estados Unidos da América (EUA)^1^, desencadeando ampla repercussão na mídia internacional especializada em saúde e nutrição. O guia é composto por: (i) um documento impresso e resumido, destinado à população em geral^1^; (ii) um vídeo institucional de aproximadamente 1 minuto e 25 segundos (https://realfood.gov/); (iii) um relatório científico com anexos^2,3^; e (iv) um apêndice detalhando porções alimentares para diferentes cenários de ingestão energética diária^4^.

Um aspecto central desse processo foi a decisão de não utilizar como base principal o relatório científico elaborado pelo *Dietary Guidelines Advisory Committee* (DGAC), publicado em dezembro de 2024 e originalmente destinado a subsidiar a formulação do guia^5^. A rejeição do relatório foi justificada pela alegação de limitações metodológicas e conceituais, incluindo o uso de um enquadramento centrado em equidade em saúde^3^. Em seu lugar, a administração responsável optou por conduzir uma revisão independente das evidências científicas, fundamentada na *expertise* de um novo comitê científico, com o argumento de que o guia deveria priorizar a melhor evidência disponível na ciência da nutrição, com foco direto na prevenção e na reversão de doenças crônicas e na promoção da saúde metabólica^3^. Cabe destacar que parte dos especialistas envolvidos nessa nova revisão declarou vínculos financeiros prévios ou atuais com as indústrias de carne, laticínios, suplementos e fórmulas infantis^6^. Embora tais relações não impliquem necessariamente viés científico, sua presença suscita questionamentos sobre potenciais conflitos de interesse na formulação das recomendações presentes no guia.

## O Que o Novo Guia Propõe?

O novo guia adota os *slogans* “Make America Healthy Again” e “Eat Real Food” (“Faça a América Saudável Novamente” e “Coma Comida de Verdade”), ancorando-se na realidade epidemiológica dos EUA, onde cerca de 70% da população apresenta sobrepeso ou obesidade, com importantes desdobramentos no aumento das doenças crônicas não transmissíveis. A justificativa apresentada pelo documento aponta para o elevado consumo de alimentos ultraprocessados, ricos em carboidratos refinados, açúcares adicionados, gorduras, sódio e aditivos químicos, associado a baixos níveis de atividade física^1^.

Nesse ponto, o guia transmite uma mensagem relevante e amplamente respaldada por evidências científicas: a necessidade de reduzir o consumo de alimentos ultraprocessados e priorizar alimentos minimamente processados^7^. Contudo, à medida que o documento avança, surgem recomendações cuja coerência com a literatura científica contemporânea se mostra menos consistente.

## Representação Gráfica de Pirâmide Alimentar: um Modelo Ultrapassado e Confuso

Um dos elementos mais controversos do novo guia é sua representação gráfica por meio de uma pirâmide alimentar invertida. Na base da pirâmide, ocupando metade do espaço, encontram-se alimentos de origem animal — incluindo carne bovina com elevado teor de gordura, frango, ovos, peixes e laticínios integrais — além de manteiga. A outra metade da base é composta por frutas, verduras e leguminosas. No topo da pirâmide, estão posicionados os cereais integrais.

O uso de pirâmides alimentares como ferramenta de orientação para a população é reconhecido como ultrapassado, por simplificar a complexidade da alimentação e não refletir o conhecimento científico atual sobre padrões alimentares, saúde e doenças crônicas^8^. Além disso, o modelo de pirâmide enfatiza quantidades e porções de grupos alimentares, quando a evidência contemporânea demonstra que a qualidade dos alimentos e os padrões alimentares como um todo são determinantes mais relevantes para a saúde^8^. Ademais, a pirâmide não contextualiza diferentes combinações alimentares nem considera a diversidade cultural e regional, aspectos centrais em guias alimentares modernos, como o Guia Alimentar para a População Brasileira^9^ e o Guia Nórdico de Nutrição^10^.

Nesse sentido, guias alimentares de diversos países, incluindo o do Brasil, substituíram a pirâmide por representações baseadas em “pratos saudáveis”, que permitem visualizar padrões alimentares equilibrados e culturalmente adaptados^9^. No caso do guia alimentar estadunidense^1^, a adoção de uma pirâmide invertida agrava ainda mais as limitações desse modelo, tornando sua interpretação menos intuitiva e potencialmente confusa para a população.

## Outros Pontos Centrais do Guia

O documento apresenta ainda as seguintes recomendações gerais^1^: (1) consumir a quantidade adequada de alimentos para cada indivíduo; (2) priorizar alimentos proteicos em todas as refeições; (3) consumir laticínios; (4) ingerir frutas e vegetais ao longo do dia; (5) incorporar gorduras consideradas saudáveis; (6) focar em alimentos integrais; (7) limitar o consumo de alimentos ultraprocessados, açúcares adicionados e carboidratos refinados; (8) limitar o consumo de bebidas alcoólicas; e (9) apresentar orientações específicas para diferentes grupos populacionais, incluindo lactentes e crianças pequenas, crianças em idade escolar, adolescentes, adultos jovens, gestantes e lactantes, idosos, indivíduos com doenças crônicas, vegetarianos e veganos.

## Ênfase na Ingestão de Proteína: Qual a Evidência Científica?

Dentre essas recomendações, destaca-se a ênfase na ingestão proteica, com valores sugeridos entre 1,2 e 1,6 g/kg/dia, a serem alcançados pre­dominantemente por meio de fontes de proteína de origem animal, como carne bovina, frango, ovos e laticínios integrais. Esses alimentos, conforme amplamente documentado, apresentam elevado teor de gordura saturada.

Tal recomendação supera de maneira significativa a Ingestão Diária Recomendada de proteína (*Recommended Dietary Allowance* — RDA)^11^ e a recomendação da Organização Mundial da Saúde^12^, fixada em 0,8 g/kg/dia para adultos saudáveis. Embora indivíduos com níveis muito elevados de atividade física possam demandar maior ingestão proteica, para a maioria da população adulta—que apresenta nível sedentário de atividade física—o consumo excessivo de proteína pode contribuir para um balanço energético positivo, favorecendo o ganho de peso e o desenvolvimento da obesidade.

Além disso, a priorização de proteína animal contrasta com estudos publicados nos últimos 10 a 15 anos, com evidências científicas consistentes que demonstram que padrões alimentares baseados em plantas (nos quais entre 50% e 80% da proteína é de origem vegetal) apresentam um melhor balanço de nutrientes e fibras e estão associados à redução do risco de doenças crônicas não transmissíveis, incluindo doenças cardiovasculares e doença renal crônica^13^. Esse aspecto é particularmente relevante para a comunidade da nefrologia, diante do crescente interesse de jovens adultos por dietas hiperproteicas voltadas ao aumento de massa muscular, que podem levar à hiperfiltração glomerular, embora os potenciais impactos adversos à saúde renal permaneçam inconclusivos^14^.

Não menos importante é o impacto ambiental dessa recomendação. Em outubro de 2025, foi publicada a atualização da dieta EAT-Lancet, um padrão alimentar proposto pela Comissão EAT-Lancet, que visa simultaneamente promover a saúde humana e a sustentabilidade ambiental, priorizando alimentos de origem vegetal e limitando o consumo de produtos de origem animal e ultraprocessados^15^. Esse documento, fundamentado em evidência científica, alerta para os riscos ambientais e de saúde associados a dietas com elevada participação de proteínas de origem animal.

Logo, pelos motivos expostos, a ênfase na ingestão recomendada de proteína nas Diretrizes Alimentares para Americanos se opõe de forma significativa a muitos guias alimentares atuais.

## Conflito Interno: Gordura Saturada Versus Padrão Alimentar Proposto

Outro ponto crítico das novas diretrizes reside em sua inconsistência interna. Enquanto o documento recomenda a priorização de alimentos proteicos em todas as refeições, ilustrada na pirâmide alimentar invertida por imagens de carnes com elevado teor de gordura e por uma ingestão proteica diária de 1,2 a 1,6 g/kg, simultaneamente orienta que a ingestão de gordura saturada não ultrapasse 10% do valor energético total da dieta. Soma-se a isso a recomendação de priorizar óleos ricos em ácidos graxos mono e polinsaturados, como o azeite de oliva, ao mesmo tempo em que manteiga e gordura de carne se apresentam como possíveis alternativas de gordura na alimentação.

Essa combinação de orientações revela uma incoerência dietética que dificulta a aplicação prática do guia. Do ponto de vista do planejamento alimentar, torna-se inviável conciliar uma dieta rica em carnes e laticínios integrais, com uso opcional de manteiga ou gordura animal como alternativas ao azeite de oliva, mantendo simultaneamente o limite recomendado de gordura saturada.

## Frutas, Hortaliças, Cereais Integrais e Microbiota Intestinal

Adicionalmente, as recomendações quantitativas para frutas e vegetais — duas porções de frutas e três de verduras por dia — situam-se abaixo do preconizado por padrões alimentares reconhecidamente associados à redução do risco de obesidade e de doenças crônicas. A posição dos cereais integrais na parte mais estreita da pirâmide, com recomendação de apenas duas a quatro porções diárias, também se afasta de modelos alimentares saudáveis amplamente respaldados pela literatura.

Finalmente, consideradas em conjunto, as quantidades propostas para carnes, laticínios, frutas, hortaliças, cereais integrais e gorduras não favorecem a manutenção de uma microbiota intestinal saudável, apesar de o próprio guia reconhecer a importância desse fator para a saúde. A ênfase em alimentos de origem animal, associada a uma ingestão relativamente baixa de fibras alimentares provenientes de frutas, hortaliças, leguminosas e cereais integrais, tende a reduzir a diversidade microbiana intestinal e a produção de metabólitos benéficos, como os ácidos graxos de cadeia curta^16^. Esse padrão alimentar pode, portanto, comprometer mecanismos relevantes para a regulação metabólica, a função imunológica e a saúde cardiovascular, contrariando evidências consolidadas que associam dietas ricas em alimentos de origem vegetal a uma maior diversidade da microbiota e a um perfil metabólico mais favorável.

## Pontos Positivos

Apesar das críticas, o guia apresenta aspectos positivos. A mensagem central de “comer comida de verdade” tem potencial para incentivar a redução do consumo de alimentos ultraprocessados e estimular o preparo de refeições em casa, o que pode trazer benefícios à saúde. A orientação para evitar cereais refinados com adição de sódio, açúcar, gorduras e aditivos químicos, bem como a recomendação de limitar bebidas açucaradas e alcoólicas, também se alinha a evidências robustas. Destaca-se ainda o material destinado à população geral, apresentado em linguagem simples e acessível.

## Relevância Para a População Brasileira

No contexto brasileiro, o impacto desse guia é irrelevante. O Guia Alimentar para a População Brasileira, publicado em 2014, é amplamente adaptado à realidade alimentar, socioeconômica e cultural do país, sendo reconhecido internacionalmente como um dos guias mais inovadores do mundo^9^. Suas recomendações são contextualizadas a partir de preparações culinárias regionais e funda­mentadas no princípio de priorizar alimentos *in natura* e minimamente processados, respeitando a sazonalidade e a cultura alimentar. Ademais, o guia brasileiro foi pioneiro ao incorporar a dimensão dos sistemas alimentares saudáveis e justos, incluindo preocupações ambientais e de equidade na produção e no acesso aos alimentos.

## Conclusão

Em síntese, as novas Diretrizes Alimentares dos EUA apresentam avanços importantes ao reconhecer o papel central dos alimentos *in natura* e minimamente processados e ao enfatizar a necessidade de reduzir o consumo de ultraprocessados em uma população marcada por altas prevalências de obesidade e doenças crônicas. No entanto, o documento também revela inconsistências internas relevantes, especialmente no que se refere à ênfase excessiva em proteína de origem animal, à representação gráfica por meio de uma pirâmide alimentar, ainda em formato invertido, o que torna sua interpretação difícil e confusa, e ao conflito entre as recomendações de ingestão proteica e o limite proposto para gordura saturada.

Ao se afastar de modelos contemporâneos baseados em padrões alimentares integrados, sustentáveis e alinhados à saúde metabólica, cardiovascular e ambiental, o guia corre o risco de limitar sua efetividade como instrumento de promoção da saúde pública. Guias alimentares desempenham papel estratégico na orientação de políticas, sistemas alimentares e escolhas individuais; por isso, sua credibilidade depende de coerência científica, viabilidade prática e alinhamento com evidências consolidadas. Nesse sentido, as novas Diretrizes Alimentares dos EUA suscitam mais questionamentos do que consensos e reforçam a importância de processos transparentes e cientificamente consistentes na formulação de recomendações dietéticas populacionais.

## Data Availability

Nenhum dado novo foi gerado ou analisado neste estudo.
